# Alternative Evolutionary Pathways for Drug-Resistant Small Colony Variant Mutants in *Staphylococcus aureus*

**DOI:** 10.1128/mBio.00358-17

**Published:** 2017-06-20

**Authors:** Sha Cao, Douglas L. Huseby, Gerrit Brandis, Diarmaid Hughes

**Affiliations:** Department of Medical Biochemistry and Microbiology, Biomedical Center, Uppsala University, Uppsala, Sweden; University of Wisconsin; Skirball Institute of Biomolecular Medicine, New York University Medical Center

**Keywords:** ATP, experimental evolution, growth compensation, hemin biosynthesis, menaquinone, SrrAB, transcriptional regulation, translational suppression

## Abstract

*Staphylococcus aureus* is known to generate small colony variants (SCVs) that are resistant to aminoglycoside antibiotics and can cause persistent and recurrent infections. The SCV phenotype is unstable, and compensatory mutations lead to restored growth, usually with loss of resistance. However, the evolution of improved growth, by mechanisms that avoid loss of antibiotic resistance, is very poorly understood. By selection with serial passaging, we isolated and characterized different classes of extragenic suppressor mutations that compensate for the slow growth of small colony variants. Compensation occurs by two distinct bypass mechanisms: (i) translational suppression of the initial SCV mutation by mutant tRNAs, ribosomal protein S5, or release factor 2 and (ii) mutations that cause the constitutive activation of the SrrAB global transcriptional regulation system. Although compensation by translational suppression increases growth rate, it also reduces antibiotic susceptibility, thus restoring a pseudo-wild-type phenotype. In contrast, an evolutionary pathway that compensates for the SCV phenotype by activation of SrrAB increases growth rate without loss of antibiotic resistance. RNA sequence analysis revealed that mutations activating the SrrAB pathway cause upregulation of genes involved in peptide transport and in the fermentation pathways of pyruvate to generate ATP and NAD^+^, thus explaining the increased growth. By increasing the growth rate of SCVs without the loss of aminoglycoside resistance, compensatory evolution via the SrrAB activation pathway represents a threat to effective antibiotic therapy of staphylococcal infections.

## IMPORTANCE

Small colony variants (SCVs) of *Staphylococcus aureus* are a significant clinical problem, causing persistent and antibiotic-resistant infections. However, SCVs are unstable and can rapidly evolve growth-compensated mutants. Previous data suggested that growth compensation only occurred with the loss of antibiotic resistance. We have used selection with serial passaging to uncover four distinct pathways of growth compensation accessible to SCVs. Three of these paths (reversion, intragenic suppression, and translational suppression) increase growth at the expense of losing antibiotic resistance. The fourth path activates an alternative transcriptional program and allows the bacteria to produce the extra ATP required to support faster growth, without losing antibiotic resistance. The importance of this work is that it shows that drug-resistant SCVs can evolve faster growth without losing antibiotic resistance.

## INTRODUCTION

*Staphylococcus aureus* is a human pathogen that causes community-associated and hospital-acquired infections. During antibiotic treatment, *S. aureus* can generate a slow-growing subpopulation of cells called small colony variants (SCVs). SCVs can cause persistent and recurrent infections that are difficult to treat and diagnose (1). The SCV phenotype can contribute to the capacity of *S. aureus* to invade and survive inside host nonprofessional immunity cells, where they avoid being killed by the immune defenses (2, 3). In addition, the SCV phenotype is associated with resistance to aminoglycosides, allowing pathogen survival against antibiotic challenge (4–6).

Environmental pressures such as antibiotic exposure can select for small colony variants (7–10). The slow-growth and aminoglycoside resistance phenotypes of *S. aureus* SCVs have been shown to be due to disruptions in the electron transport chain (5, 11, 12). Defects in the respiratory chain result in decreased cross-membrane potential. As a consequence of this reduced potential, the influx of aminoglycoside antibiotics is also reduced, and this results in reduced susceptibility of SCV strains to aminoglycoside antibiotics. An additional effect of the reduction of cross-membrane potential is decreased ATP production, which accounts for the slow-growth phenotype of these strains. In agreement with this, the majority of mutations that cause the SCV phenotype are found in genes involved in the biosynthesis of menadione and hemin, two compounds required in the biosynthesis of the electron transport chain components menaquinone and cytochromes, respectively (13–15).

The inhibition of respiratory pathways by SCV mutations in menadione and hemin biosynthesis genes will produce effects on bacterial physiology similar to those experienced in a low-oxygen environment. Accordingly, SCV mutations can be expected to cause upregulation of genes involved in anaerobic metabolism as an adaptive response. In *Bacillus subtilis*, where the regulation of anaerobic metabolism is best understood, adaptation depends on the interplay between a membrane-bound oxygen-sensitive two-component regulator, ResDE, a redox regulator, Frn, involved in nitrate respiration, and a redox-sensing repressor, Rex, that responds to changes in the ratio of NADH to NAD^+^ (16–19). *S. aureus* has a similar set of regulators, with SrrAB, a membrane-bound two-component system being homologous to ResDE in *B. subtilis*. In *B. subtilis*, Rex regulates genes encoding components of the respiratory chain, whereas genes involved in anaerobic metabolic pathways are regulated mainly by Frn and ResDE (16–20). In contrast, in *S. aureus*, expression of the respiratory chain component genes appears to be Rex independent while genes involved in anaerobic metabolism and fermentation are regulated by Rex. Intriguingly, in *S. aureus* Rex is also a negative effector of SrrA synthesis (20).

Growth-compensatory mutations occur at a high frequency for SCVs of *S. aureus* and can mask the initial slow-growth phenotype. In the clinical setting, the phenotypic instability of SCVs increases the probability that they avoid detection, increasing the risk of applying an inappropriate antibiotic therapy (5). We have previously reported that the slow growth of SCVs can be compensated for by reversion of the mutated hemin and menadione biosynthesis genes, either to the wild-type gene sequence, or by intragenic suppression, the acquisition of a second-site mutation within the mutated gene causing the SCV phenotype (21, 22). Isolates that carry intragenic suppressor mutations have completely or partially restored the function of their respiratory chain and therefore have restored sensitivity to aminoglycosides associated with their improved growth phenotype.

In this study, we investigated if there are alternative evolutionary pathways that can reverse the slow-growth phenotype of *S. aureus* SCVs. We were particularly interested to determine if there was an evolutionary path to increase growth rate without restoration of respiratory chain activity. The prediction was that if mutants could compensate for the SCV phenotype in this manner, they would be expected to retain their reduced susceptibility to aminoglycoside antibiotics and consequently would pose a threat to effective antibiotic therapy in the clinical setting. A recent study found mutants with these characteristics, but so far, no functional basis for the suppression has been reported (23). Here we describe the underlying mechanism of multiple extragenic evolutionary pathways that are open to SCVs to suppress the slow-growth phenotype. One of these evolutionary pathways allows the SCVs to restore fast growth while retaining their antibiotic resistance phenotype.

## RESULTS

### Selection and characterization of *S. aureus* small colony variants.

A total of 107 independent kanamycin-resistant small colony variants (SCVs) of *S. aureus* were selected and purified (Materials and Methods). It was previously shown that *S. aureus* SCVs are defective in respiration and generally show auxotrophy for hemin or menadione (5, 14, 21, 24, 25). In agreement with this, most of the selected SCV isolates were auxotrophic for either hemin (48/107) or menadione (58/107), and only one isolate showed no auxotrophy for either hemin or menadione. Growth rates were determined for all 107 isolated SCVs. In general, hemin-auxotrophic SCVs grew faster than menadione-auxotrophic SCVs (*P* < 0.001), with mean growth rates (relative to the parental wild type) of 0.37 ± 0.05 and 0.28 ± 0.04, respectively (see Fig. S1 in the supplemental material). Based on differences between individual SCV mutants, 26 mutants were chosen for whole-genome sequencing (WGS) to identify mutations associated with the SCV phenotypes. The criteria for choosing the specific mutants for sequencing were that they should include representatives of each of the different auxotrophic phenotypes identified, mutants with different growth rates around the median value measured for each auxotrophy, and mutants that covered the full range of kanamycin MIC values measured. The sequenced mutants included 10 hemin auxotrophs, 15 menadione auxotrophs, and the single nonauxotrophic mutant. The sequence analysis (see Table S1 in the supplemental material) showed that each of the menadione-auxotrophic SCVs carried a mutation in one of the menadione biosynthesis genes (*menA*, *menB*, or *menE*) and that each of the hemin-auxotrophic SCVs carried a mutation in one of the hemin biosynthesis genes (*hemB*, *hemC*, *hemD*, *hemE*, or *hemH*). The single nonauxotrophic SCV carried mutations in *qoxB*, *oxaA*, *srrA*, and *ftsQ*, genes that are involved in respiration or cell division (26–31), possibly explaining the SCV phenotype of this mutant. The median kanamycin MIC of the 26 mutant strains is 48 mg/liter (Table S1). In 6 of the strains, there was an additional mutation in a gene previously associated with aminoglycoside resistance, *fusA* or *rplF* (4, 21), and in these mutants, the MIC was higher than average, at 64 to 256 mg/liter (Table S1). In 10 of the 26 SCVs, the only acquired mutation in the genome was a single mutation in one of the genes in either the menadione or hemin biosynthesis pathways, providing strong evidence that single mutations in either of these pathways are sufficient to account for the kanamycin-resistant SCV phenotype.

10.1128/mBio.00358-17.1 FIG S1  Relative growth rates of menadione-auxotrophic, hemin-auxotrophic, and nonauxotrophic SCVs relative to the wild-type growth rate (set at 1). Asterisks indicate that the relative growth rates of menadione-auxotrophic and hemin-auxotrophic SCVs differ significantly at the 99.9% confidence level. Download FIG S1, PDF file, 0.1 MB. Copyright © 2017 Cao et al. 2017 Cao et al. This content is distributed under the terms of the Creative Commons Attribution 4.0 International license.

10.1128/mBio.00358-17.3 TABLE S1  Genotypes and phenotypes of 26 whole-genome-sequenced SCVs. Download TABLE S1, PDF file, 0.1 MB. Copyright © 2017 Cao et al. 2017 Cao et al. This content is distributed under the terms of the Creative Commons Attribution 4.0 International license.

### Selection of growth-compensated mutants.

Growth-compensatory mutants were selected from four hemin-auxotrophic and four menadione-auxotrophic SCVs (Table 1). The SCVs chosen for selection included four mutants with single amino acid substitutions, three with nonsense mutations, and one with a frameshift mutation, to represent a variety of distinct mutant types. Between 10 and 34 independent lineages of each of the 8 different SCVs were evolved for improved growth. After 150 generations of growth, all evolved lineages contained faster-growing mutants, and from each lineage a single growth-compensated mutant was selected for further analysis (for a total of 140 independently evolved growth-compensated mutants). Each of the evolved mutants was initially analyzed by measuring growth rate, growth yield, and MIC of kanamycin and by DNA sequencing of the relevant *hem* or *men* mutation associated with the SCV phenotype (see Table S2 in the supplemental material). Mutants that had acquired a reversion to the wild-type sequence or had acquired a second-site mutation in the relevant *hem* or *men* gene were not analyzed further. Such mutants carrying reversions or intragenic mutations had in every case an increase in growth rate and/or growth yield, associated with a decrease in the MIC of kanamycin (Table S2). However, in 38 of the sequenced isolates, originating from four different SCVs, increased growth rate and/or growth yield had occurred without the acquisition of an intragenic mutation in the relevant *hem* or *men* gene (Table 1). Genomic DNA was prepared from each of these 38 mutants, and their complete genotypes were determined by whole-genome sequencing. The WGS data revealed the presence of at least one putative extragenic suppressor mutation in each of these mutants (Table 1; Table S2). The functional distribution of these extragenic mutations was highly nonrandom: 28 mutants had a tRNA gene mutated, 3 mutants had a mutation affecting *prfB*, 1 had a mutation in *rpsE*, and 6 had mutations in *srrA* or *srrB* (Table S2). More than half of the 38 mutants carried only a single extragenic mutation, strongly supporting the role of individual mutations in tRNAs, *prfB*, *rpsE*, or *srrAB* as sufficient to cause the suppression of the SCV phenotype. According to the known functions of these mutated genes, the extragenic suppressors could be divided into two major groups: those presumed to act at the level of mRNA translation (tRNAs, ribosomal protein S5, and RF-2) and those presumed to act at the level of transcriptional regulation (SrrAB).

10.1128/mBio.00358-17.4 TABLE S2  Genotypes and phenotypes of SCVs and growth-compensated mutants after selection. Download TABLE S2, PDF file, 0.2 MB. Copyright © 2017 Cao et al. 2017 Cao et al. This content is distributed under the terms of the Creative Commons Attribution 4.0 International license.

**TABLE 1:** Selection by serial passage of SCVs for faster growth

Strain	Genotype^a^	No. of independent evolved lineages	No. of reversion or intragenic mutations	No. of lineages with extragenic mutations	Class of genes with extragenic mutations (*n*) identified by WGS^b^
AH671	*hemC* E70fs	10	10		
AH646	*hemC* D74Y	12	12		
AH727	*hemC* D104Y	22	22		
AH868	*hemH* K158^UAA^	20	9	11	tRNA Ser (4), tRNA Tyr (5), ribosomal protein *rpsE* (1)
AH847	*menA* W15^UGA^	10	7	3	Release factor 2 *prfB* (3)
AH855	*menB* D98G	10	10		
AH875	*menB* D151N	34	26	8	tRNA Asp (2), *srrA* (1), *srrB* (5)
AH635	*menE* Y31^UAA^	22	6	16	tRNA Gln (5),^c^ tRNA Glu (1), tRNA Leu (1), tRNA Lys (4),^c^ tRNA Ser (4), tRNA Tyr (3)

a The *hem* or *men* mutations indicated are the full genotype of each SCV, with the exception of AH847 (*menA* W15^UGA^ SAOUHSC-00698 L319S) and AH855 (*menB* D98G *fusA* W120L SAOUHSC-02417 A114S).

b Some of the evolved strains had acquired 1 to 3 additional extragenic mutations (see Table S2 for details). The number in parentheses (*n*) indicates the number of independent isolates of mutations affecting each gene class.

c Two of the evolved strains had each acquired mutations affecting two different tRNA genes (see Table S2 for details).

### SCV suppressor mutation affecting mRNA translation.

Three different classes of SCV suppressor mutations were isolated in genes of the translational machinery, affecting tRNAs, ribosomal protein S5, or RF-2. In 20 of the 32 evolved strains with mutations in these genes, the respective mutation was the only mutation in the genome in addition to the SCV mutation. These 20 isolates included all of those with mutations in S5 and RF-2 and at least one example of each affected tRNA type (Table S2). This argues strongly that the observed suppression is fully explained by the altered activity of the affected tRNAs, S5, and RF-2.

Mutations in tRNA genes were selected as suppressors of two SCVs caused by UAA nonsense mutations (*hemH* K158^UAA^ and *menE* Y31^UAA^). In total, 26 independent compensatory mutations in 12 distinct tRNA genes that encode 6 different amino acids were identified for the two strains carrying UAA nonsense codons. Each of these mutations changed the anticodon of the respective tRNA to 3′-UUA-5′, which is expected to lead to translational suppression of the ochre stop codon and restored function of the mutated *hemH* and *menE* genes (Table S2). The third SCV that acquired tRNA suppressor mutations carried the SCV-mutation *menB* D151N^GAU to AAU^. Two compensatory mutations were isolated for this strain that changed the anticodon of either of two aspartate tRNA genes to 3′-AUU-5′. This mutation would allow the asparagine codon at position 151 in the mutated *menB* gene to be decoded as an aspartate and thus restore the wild-type protein sequence. The tRNA suppressor mutations increased the exponential growth rate and/or final cell yield and at the same time reduced the MIC of kanamycin (Table S2). The MICs of the suppressed strains were not uniformly reduced to the wild-type level, but this is not unexpected because most of the tRNA suppressor mutations would not restore the original amino acid sequence of the mutated protein and the specific activity of the mutated tRNAs might also differ from that of the equivalent wild-type species.

One of the evolved *hemH* K158^UAA^ isolates acquired the mutation *rpsE* V105F. Ribosomal protein S5 plays an important role in translational fidelity. In *Bacillus subtilis*, a mutation in *rpsE* at position G104 was reported to induce high-level readthrough of a UGA stop codon (32). This amino acid position corresponds to *rpsE* V105 in *S. aureus*. It is therefore very likely that the *rpsE* V105F mutation leads to a readthrough of the *hemH* K158^UAA^ stop codon leading to the production of active HemH protein. The *rpsE* V105E mutation increased the final growth yield of the mutant and significantly decreased the kanamycin MIC from 48 to 8 mg/liter (Table S2).

The final class of translational suppressor mutations were found in *prfB*, the gene for protein release factor 2 (RF-2). These mutations were exclusively found in strains that carried an opal stop codon in *menA* (*menA* W15^UGA^). RF-2 is the only release factor that recognizes the UGA stop codon to terminate mRNA translation in *S. aureus* (33). The suppressor mutations in RF-2 most likely lead to a readthrough of the internal *menA* stop codon, explaining the increased final cell yield and decreased kanamycin MIC (from 48 down to 1.5 to 24 mg/liter) in the evolved strains (Table S2).

Taken together, these data support the hypothesis that one evolutionary path leading to growth compensation of SCV mutants is by the acquisition of translational suppressor mutations that restore, either completely or partially, the functionality of mutated genes involved in menadione and hemin biosynthesis. This evolutionary path leads to the recovery of the respiratory chain, improving cellular growth, but at the same time reduces the level of resistance to aminoglycosides.

### SCV suppressor mutations affecting the SrrAB two-component transcriptional regulator.

The second group of extragenic suppressors of the SCV phenotype carried single amino acid substitution mutations in *srrA* or *srrB* (Table 1; Table S2). SrrAB (staphylococcal respiratory response) is a two-component system that acts in the global transcriptional regulation of virulence factors and contributes to its survival in anaerobic environments (26, 28, 34). In each strain carrying these putative SCV suppressor mutations in *srrAB*, the exponential growth rate was significantly increased, but the MIC for kanamycin also remained high (Table S2). In one of the 6 evolved strains, the mutation *srrB* S386L is the only mutation in the genome in addition to the SCV *menB* mutation, arguing strongly that the observed suppression is fully explained by the altered activity of SrrB.

In this evolution experiment, transcriptional suppressor mutations in *srrAB* were only isolated for one of the SCV mutants (*menB* D151N), leaving open the possibility that this class of suppressors is specific to this particular SCV mutation. Alternatively, there might simply be a larger mutational target size for translational suppressors (multiple tRNA genes, translation factors, and ribosomal proteins) explaining their greater frequency. To address the possibility that SCV suppressor mutations in *srrAB* might be unique to *menB* D151N, we selected growth-compensatory mutants by serial passage for several additional kanamycin-resistant SCV mutants. Since the initial *srrAB* mutations were found in a menadione auxotroph, we decided to evolve five additional menadione auxotrophs. We also evolved one hemin auxotroph to test if *srrAB* mutation could also be selected for in this background. This evolution was done in the presence of kanamycin (to reduce the probability of selecting reversions, intragenic mutations, and translational suppressors, which were all predicted to reduce the MIC of kanamycin). Five independent lineages of each of the six SVCs were evolved by serial passage for 150 generations. At the endpoint, each evolved culture was tested by spreading 200 CFU on agar containing kanamycin and screening for the presence of faster-growing colonies. Faster-growing colonies were recovered from 7 of the 30 independent cultures (from 4 different SCVs, including from the hemin-auxotrophic SCV). The *srrAB* genes were sequenced, and mutations in either of these genes were found in 5 of the evolved strains, including mutants evolved from the hemin-auxotrophic SCV (Table 2). These results show that amino acid substitution mutations in *srrAB* represent a general mechanism that compensates for the SCV phenotype, with maintenance of kanamycin resistance, for both hemin-auxotrophic and menadione-auxotrophic SCVs (Table 2).

**TABLE 2:** Selection of growth-compensatory mutations in *srrAB*^a^

Parental strain (and evolved strain[s])	SCV mutation	*srrAB* mutation	Mean fitness ± SD	Kan MIC
AH704	*menA* E72K		0.28 ± 0.01	64
AH631	*menB* G81*		0.25 ± 0.01	64
AH846	*menB* D98G		0.27 ± 0.02	48
**AH1560**	***menB* D98G**	***srrB* T373K**	**0.53 ± 0.02**	**48**
AH841	*menB* G121D		0.29 ± 0.01	64
**AH1576**	***menB* G121D**	***srrB* T373K**	**0.38 ± 0.00**	**48**
AH635	*menE* Y31*		0.28 ± 0.02	32
**AH1570**	*menE* Y31*	***srrA* M55I**	**0.41 ± 0.01**	**32**
AH839	*hemE* A211fs		0.36 ± 0.01	48
**AH1643**	***hemE* A211fs**	***srrB* A250T**	**0.57 ± 0.01**	**48**
**AH1647**	***hemE* A211fs**	***srrB* A487T**	**056 ± 0.00**	**48**

a Growth-compensatory mutations were selected by serial passage of 5 lineages of each of 6 SCV parental strains for 150 generations in LB supplemented with 15 mg/liter kanamycin. Growth compensation occurred in 7 lineages. Mutations in *srrA* or *srrB* were identified in 5 of these lineages. (Genotypes and phenotypes of the evolved strains are shown in boldface.) All growth-compensated mutants grow significantly faster than the respective parental strains (*t* test, *P* < 0.001).

### Effects of SCV suppressor mutations on membrane potential.

**FIG 1:**
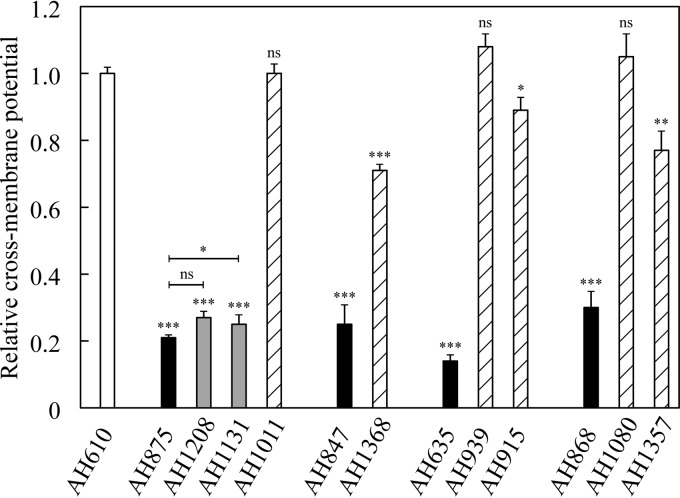
Relative cross-membrane potential of the wild type, SCVs, and growth-compensated mutants. All values are relative to the cross-membrane potential of the wild type (white). Relative cross-membrane potentials of menadione-auxotrophic SCVs (AH875, AH847, and AH635) and hemin-auxotrophic SCV (AH868) are shown in black. Isogenic evolved mutants that additionally carry transcriptional SCV suppressor mutations affecting *srrB* (AH1131) and *srrA* (1028) are shown in gray, and translational SCV suppressor mutations affecting tRNA genes (AH1011, AH939, AH915, and AH1080), RF2 (AH1368), or S5 (AH1357) are hatched. Unless otherwise indicated, all significance values (*t* test) are compared to the wild-type cross-membrane potential. ns, nonsignificant; *, *P* < 0.05; **, *P* < 0.01; ***, *P* < 0.001.

Mutations in the menadione and hemin biosynthesis genes reduce the production of precursors for the biosynthesis of menaquinone (menadione) and cytochromes (hemin), two components of the electron transport chain. This leads to a decreased cross-membrane potential in small colony variants, resulting in a decreased uptake of cationic compounds, with reduced susceptibility to aminoglycosides as a clinically significant effect (5). A striking difference between the two classes of extragenic SCV suppressors was that in general the translational suppressors restored susceptibility to kanamycin, whereas the *srrAB* suppressors did not. We asked whether this phenotypic difference in effect between these two classes of SCV suppressor mutations could be explained by their different effects on cross-membrane potential. The membrane potential was measured (Materials and Methods) on a set of representative strains, including the wild type, 4 different SCV mutants, and 8 strains carrying different SCV suppressor mutations (Fig. 1). As expected, all SCVs had a very low cross-membrane potential: between 0.14 and 0.30 relative to the wild type (Fig. 1). The acquisition of a translational suppressor mutation, affecting tRNAs, ribosomal protein S5, or RF-2, restored the cross-membrane potential back to wild-type or near-wild-type levels (0.71 to 1.08). In contrast, the transcriptional suppressor mutations (SrrAB) caused only a minor increase in the cross-membrane potential from 0.21 in the SCV to 0.25 to 0.27 in the compensated mutants. The different effects of each class of suppressor mutation on membrane potential show that the transcriptional and translational suppressor mutations represent two distinct pathways to suppress the SCV phenotype. We conclude that the SCV suppressor mutations in *srrAB* compensate for the slow-growth phenotype without restoring the cross-membrane potential, which implies that cellular respiration is not restored. This is consistent with their retention of a kanamycin resistance phenotype.

### Inactivation of *srrAB* does not suppress the SCV phenotype.

Only missense mutations in *srrA* or *ssrB* were isolated in this study, and no frameshift or nonsense mutations were observed. Additionally, both mutations isolated in *srrA* were identical (*srrA* M55I), and the majority of mutations in *srrB* (5 out of 9) were located in the phosphoacceptor region of SrrB (N366 to E583) (http://www.uniprot.org) (Table 2; Table S2). These biases in type and location of the compensatory mutations argue that they cause an activation of the SrrAB regulatory system rather than an inactivation. To test this hypothesis, an *srrAB* deletion was moved into the SCV strain with the *menB* D151N mutation. Growth data and kanamycin MIC measurements showed that the deletion of *srrAB* had no effect on the phenotype of the SCV strain, showing that inactivation of the SrrAB system has no compensatory effect on the SCV mutant (Table 3). We concluded that the SCV suppressor mutations in *srrAB* are not inactivating mutations.

**TABLE 3:** Relative fitness and kanamycin MIC of SCV mutant and isogenic *srrAB* mutant strains

Strain	SCV mutation	*srrAB* mutation	Mean fitness ± SD	Kan MIC (mg/liter)
AH875	*menB* D151N		0.29 ± 0.01	48
AH1208	*menB* D151N	*srrA* M55I	0.48 ± 0.02	32
AH1131	*menB* D151N	*srrB* V420D	0.48 ± 0.02	48
AH1670	*menB* D151N	*srrAB* deletion^a^	0.23 ± 0.02	48

a *srrAB*<>Em^r^.

### SCV suppressor mutations in *srrAB* activate the SrrAB system.

Since the compensatory mutations apparently do not inactivate the SrrAB system, it is most likely that they lead to constitutive activation of the two-component system. Alternatively, the *srrAB* mutations might downregulate SrrA activity without abolishing it. To distinguish between these two possibilities mRNA levels of two genes (*pflB* and *adhE*) that are regulated by SrrA (28, 35) were determined by quantitative PCR (qPCR). As expected, deletion of *srrAB* leads to a 13-fold reduction in mRNA levels of *pflB* and a 3-fold reduction of *adhE* (see Table S4 in the supplemental material). In contrast, mRNA levels of *pflB* and *adhE* increased 6-fold to 7-fold in strains that carried the *srrA* M55I or *srrB* V420D mutation (Table S4). These data indicate that the *srrAB* mutations lead to constitutive activation of the two-component system.

**FIG 2:**
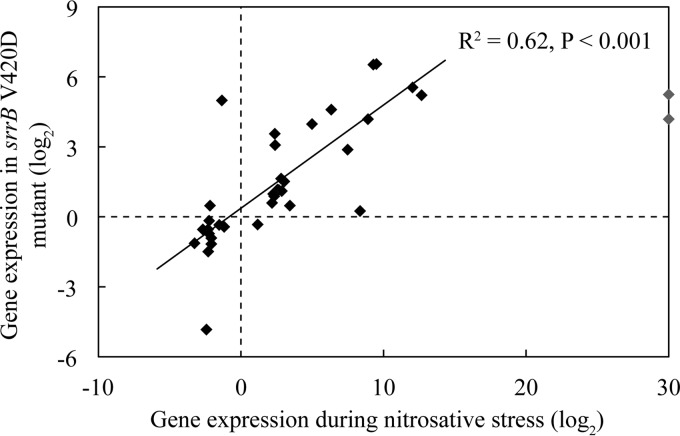
Correlation in the patterns of gene expression associated with SCV suppressor mutation affecting SrrAB and nitrosative stress. Shown is a comparison of the pattern of gene expression associated with the SCV suppressor mutation *srrB* V420D with that previously associated with nitrosative stress (28). Two outliers (*nrdD* and *nrdG*) are indicated in gray.

It has previously been shown that the SrrAB system is activated by nitrosative stress (28). To further test the hypothesis that SCV-compensatory mutations in *srrAB* cause constitutive activation of SrrAB, transcriptome sequencing (RNA-seq) analysis was conducted on a set of isogenic strains (wild type, SCV *menB* D151N, and isogenic strains carrying the SCV suppressor mutation *srrA* M55I or *srrB* V420D). The RNA-seq analysis (Table S4) showed that the SCV-compensatory mutations in *srrA* and in *srrB* each had similar effects on global gene expression (*R*^2^ = 0.69, *P* < 0.001) (see Fig. S2 in the supplemental material). The effects of these mutations also mimic the effect of nitrosative stress (*R*^2^ = 0.62, *P* < 0.001), in activating the system, in agreement with the SCV suppressor mutations causing activation of the SrrAB system (Fig. 2).

10.1128/mBio.00358-17.2 FIG S2  Effects of *srrA* and *srrB* mutations on global gene expression. Shown is a comparison of global gene expression levels between *srrA* M55I and *srrB* V420D mutants. The solid line indicates linear correlation. Download FIG S2, PDF file, 2.8 MB. Copyright © 2017 Cao et al. 2017 Cao et al. This content is distributed under the terms of the Creative Commons Attribution 4.0 International license.

Even though the precise mechanism by which SrrAB is activated is unknown, it is thought that it involves sensing of an overreduced menaquinone pool (28). Menadione-auxotrophic SCVs do not produce any menaquinone, so the SrrAB system should be inactive. In contrast, the hemin-auxotrophic SCVs should have a highly reduced menaquinone pool due to their lack of cytochromes. To test this hypothesis, RNA-seq analysis was conducted on a hemin-auxotrophic SCV (*hemD* Q107fs). The results show SrrAB-activated genes are highly transcribed in the hemin-auxotrophic SCV, indicating that the SrrAB system is indeed activated (Table 4; see Table S3 in the supplemental material). This might also explain why hemin-auxotrophic SCVs grow faster than menaquinone-auxotrophic SCVs (Fig. S1). Nevertheless, mRNA expression levels of *pflB* and *adhE* are 4-fold to 8-fold higher in menaquinone-auxotrophic SCVs with an *srrAB* mutation than in the hemin-auxotrophic SCV (*P* < 0.001) (Table 4; Table S3), and acquisition of an *srrAB* mutation in a hemin-auxotrophic SCV leads to a significant increase in fitness (Table 2). These data indicate that the SrrAB system is partly activated in hemin-auxotrophic SCVs, leading to increased growth (compared to menadione-auxotrophic SCVs), but further constitutive activation will provide a significant growth benefit.

10.1128/mBio.00358-17.5 TABLE S3  Relative gene expression measured by qPCR, comparing the effect of SCV suppressor mutations in *srrAB* with the effect of a deletion of *srrAB*. Download TABLE S3, PDF file, 0.1 MB. Copyright © 2017 Cao et al. 2017 Cao et al. This content is distributed under the terms of the Creative Commons Attribution 4.0 International license.

**TABLE 4:** Mutations in *menB*, *srrA*, and *srrB* increase mRNA level of genes in fermentation pathways for ATP production

Gene no., name	Gene function	Gene expression (log_2_ ratio)			
		Men			Hem (SCV vs WT)^d^
		SCV vs WT^a^	*srrA* vs SCV^b^	*srrB* vs SCV^c^	
Pyruvate fermentation					
SAOUHSC_00113, *adhE*	Alcohol dehydrogenase	2.1	3.2	3.3	1.6
SAOUHSC_00608, *adh1*	Alcohol dehydrogenase I	3.1	3.3	3.6	3.5
SAOUHSC_00206, *ldh1*	l-Lactate dehydrogenase	6.8	−0.2	−0.3	3.4
SAOUHSC_02922, *ldh2*	l-Lactate dehydrogenase	−0.6	2.9	2.8	0.8
SAOUHSC_00188, *pflA*	Pyruvate formate-lyase 1 activating enzyme	1.4	6.5	6.6	6.1
SAOUHSC_00187, *pflB*	Formate acetyltransferase	1.5	6.5	6.5	6.2
Amino acid fermentation					
SAOUHSC_01451, *ilvA1*	Threonine dehydratase	2.4	5.7	6.2	5.8
SAOUHSC_01452, *ald1*	Alanine dehydrogenase	2.8	5.7	6.1	6.1
SAOUHSC_02839	l-Serine dehydratase	0.5	2	1.2	0.4
SAOUHSC_02840	l-Serine dehydratase	0.4	2.2	1.5	0.5
Arginine deiminase pathway					
SAOUHSC_02969, *arcA*	Arginine deiminase	10.2	−0.8	−0.8	7.3
SAOUHSC_01128, *arcB1*	Ornithine carbamoyltransferase	1.0	6.2	5.6	4.2
SAOUHSC_02968, *arcB2*	Ornithine carbamoyltransferase	10.1	−0.8	−0.7	7.3
SAOUHSC_01129, *arcC1*	Carbamate kinase	0.8	5.3	4.9	3.2
SAOUHSC_02965, *arcC2*	Carbamate kinase	8.5	−0.6	−0.7	5.8
SAOUHSC_02967, *arcD*	Arginine/ornithine antiporter	9.6	−0.7	−0.6	6.9
SAOUHSC_02964, *argR*	Transcriptional regulator	8.4	−0.6	−0.6	5.8
Peptide transport genes					
SAOUHSC_01450 *potE*	Amino acid permease	1.8	5.7	6.1	5.2
SAOUHSC_02763	Peptide ABC transporter	−3	3.2	3.2	−0.1
SAOUHSC_02764	Peptide ABC transporter	−3.2	3.4	3.3	−0.1
SAOUHSC_02766	Peptide ABC transporter	−2.3	2.9	2.9	−0.1
SAOUHSC_02741	Amino acid ABC transporter permease	2.5	0.9	0.9	1.5
SAOUHSC_02767	Peptide ABC transporter	−3.0	3.7	3.9	0.0

a SCV mutant AH875 (*menB* D151N).

b Growth-compensated SCV mutant AH1208 (*menB* D151N *srrA* M55I).

c Growth-compensated SCV mutant AH1131 (*menB* D151N *srrB* V420D).

d SCV mutant AH862 (*hemD* Q107fs).

Taken together, these data support the conclusion that the SCV-compensatory mutations selected in *srrAB* lead to the constitutive activation of the SrrAB two-component system.

### SCV suppressor mutations in *srrAB* open alternative pathways to produce ATP.

Menadione- and hemin-auxotrophic SCVs grow slowly because they have a defective electron transport chain (5). The mutations in these pathways cause a decrease in proton motive force and consequently reduce ATP production from ATP synthase. Published data show that SCV mutants compensate phenotypically for the loss of ATP from respiration by increasing the expression of genes involved in the arginine deiminase and the pyruvate fermentation pathways (36, 37). The upregulation of these pathways increases the production of ATP from arginine and pyruvate in the SCV mutants and is believed to be important for supporting their ability to grow, albeit slowly, given that they are defective in producing ATP by the respiratory pathway (5). We asked whether the mutations in *srrAB* that increase the growth of SCV mutants also affected the expression of genes in the arginine deiminase and/or pyruvate fermentation pathways. The global RNA-seq analysis (Table S4) showed that the SCV mutant (*menB* D151N), as expected, had significantly increased expression of the genes in both pathways, consistent with the published data (36, 37). Importantly, RNA-seq analysis on the isogenic SCV suppressor strains carrying either *srrA* or *srrB* showed a significant further increase in the expression levels of several of the genes in each of these pathways (Table 4). Cellular ATP levels were measured for a set of strains (wild type, SCV *menB* D151N, and an isogenic strain carrying the SCV suppressor mutation *srrB* V420D) to test the hypothesis that *srrAB* mutations lead to increased ATP production. As expected, cellular ATP levels were very strongly reduced in the SCV strain (0.36 ± 0.05 relative to wild type) and partly restored in the strain that carried the *srrB* mutation (0.53 ± 0.01 relative to wild type). These data are consistent with these suppressor mutants having an increased upregulation of these pathways to support increased production of ATP production associated with their increased growth.

10.1128/mBio.00358-17.6 TABLE S4  RNA-seq analysis. Download TABLE S4, XLSX file, 0.6 MB. Copyright © 2017 Cao et al. 2017 Cao et al. This content is distributed under the terms of the Creative Commons Attribution 4.0 International license.

### SCV suppressor mutations in *srrAB* increase the ability to utilize amino acids to support growth.

**FIG 3:**
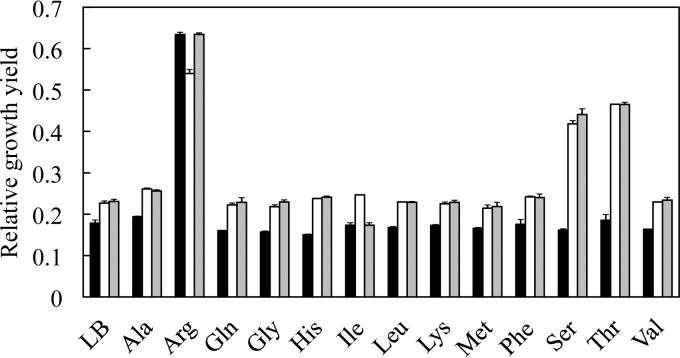
Addition of particular amino acids to growth medium specifically increases the growth yield of an SCV mutant. Shown are relative growth yields of the SCV mutant AH875 (*menB* D151N [black]), and isogenic evolved strains carrying growth-compensatory mutations in *srrA* or *srrB*: AH1208 (*srrA* M55I [white]) and AH1131 (*srrB* V420D [gray]). Strains were grown in LB with the addition of the individual amino acids indicated. All values are growth yield relative to the growth yield of the wild type in LB.

The SCV mutants were evolved for faster growth in LB medium, in which small peptides and amino acids are the most important energy source for the bacteria. We asked whether genes involved in peptide or amino acid transport and fermentation were also upregulated in the growth-compensated *srrAB* mutants. The RNA-seq data showed that genes encoding four different peptide ABC transporters, and *potE*, encoding an amino acid permease, were upregulated between 8-fold and 67-fold, while four enzymes involved in the fermentation of alanine, serine, or threonine were upregulated 2-fold to 73-fold above the level in the SCV mutant (Table 4). We asked whether the addition of amino acids to the growth medium would preferentially increase the growth rate of the strains carrying the *srrAB* mutations, relative to the effect of such medium additions on the original SCV mutant. This was to test the hypothesis that these increased mRNA levels support an improved ability, associated with the *srrAB* mutations, to transport and utilize peptides and amino acids. To test this hypothesis, SCVs with and without *srrAB* mutations were grown in LB medium supplemented with one of 13 different l-amino acids. Most of the added amino acids had no major effect on growth rate (Fig. 3). The exceptions were arginine, serine, and threonine. The addition of arginine improved the growth of the SCV even in the absence of any *srrAB* mutation (Fig. 3). The growth improvement in response to arginine was not unexpected because genes in the arginine deiminase pathway are strongly upregulated in the SCV mutant, and the mRNA levels remain high in the *srrAB* SCV suppressor mutants (Table 4). In contrast, the addition of either serine or threonine significantly improved the final growth yield of strains carrying SCV suppressor mutations in either *srrA* or *srrB* but not that of the parental SCV strain (Fig. 3). This strain-specific increase in growth yield associated with the addition of these amino acids is consistent with the transcriptional patterns revealed in the RNA-seq analysis where the mRNA levels of several genes involved in serine and threonine fermentation are significantly increased in strains with these mutations in *srrAB* (Table 4). For example, the product of *ilvA* (threonine dehydratase), the level of which is increased more than 50-fold by each of the *srrAB* mutations, can degrade both threonine and serine to pyruvate (38), which can then be further fermented to generate ATP. In addition to its role in utilization of threonine for carbon and energy, upregulation of *ilvA* will aid in the recycling of reduced NADPH cofactor via the *ilvBCD* pathway (39).

**FIG 4:**
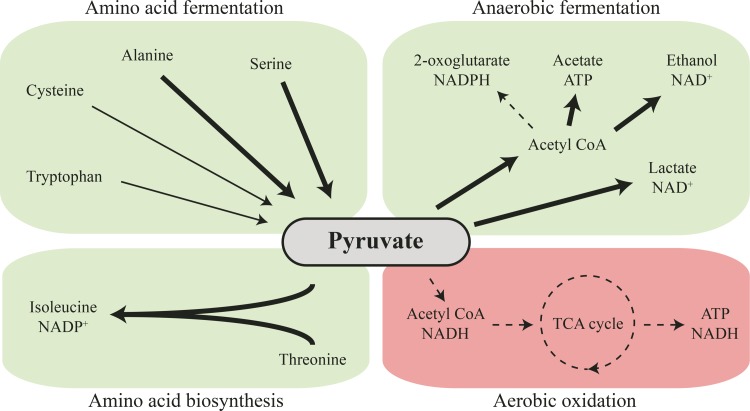
Global changes in cellular metabolism caused by activation of the SrrAB two-component system. Activation of the SrrAB two-component system causes a shift from aerobic oxidation toward anaerobic fermentation. Pathways that are repressed are shown by dashed arrows, and pathways that are overexpressed are shown by bold arrows.

In summary, the major phenotypic effect of the *srrAB* mutations is to increase the expression of genes involved in amino acid import and fermentation and of genes involved in pyruvate fermentation, potentially allowing the SCV mutants to increase their ATP production in the absence of a functioning respiration pathway (Fig. 4). This bypass mechanism of suppression of the SCV phenotype explains how the growth rate in the *srrAB* mutants could be improved without affecting their membrane potential and explains how they continue to maintain resistance to kanamycin.

### The kanamycin resistance phenotype of SCV suppressor mutations in *srrAB* is stable within the population.

The SCV suppressor mutations in *srrAB* do not restore growth to wild-type levels. Therefore, additional mutations might be selected in the course of evolution to further increase growth. If these mutations were to restore the cellular cross-membrane potential, it would lead to loss of kanamycin resistance. In that case, the kanamycin resistance phenotype of the SCV suppressor mutations in *srrAB* would only represent a transient state in the evolution of small colony variants. To test this possibility, we evolved five isolates with SCV suppressor mutations in *srrAB* for increased growth. Five lineages were evolved for each isolate (25 lineages in total), and kanamycin MICs were determined after 30 generations of growth. None of the 25 populations showed a significant decrease in kanamycin MIC (see Table S5 in the supplemental material). We conclude that the kanamycin resistance phenotype is stable within the populations.

10.1128/mBio.00358-17.7 TABLE S5  MICs of populations with SCV suppressor mutations in *srrAB* after 30 generations of evolution. Download TABLE S5, PDF file, 0.1 MB. Copyright © 2017 Cao et al. 2017 Cao et al. This content is distributed under the terms of the Creative Commons Attribution 4.0 International license.

## DISCUSSION

Small colony variants of *S. aureus* can cause persistent, recurrent, and antibiotic-resistant infections that are difficult to diagnose and treat. Strains isolated from patients might not display the SCV phenotype because growth-compensatory evolution is common. Understanding the types of compensatory mutations that are acquired during the evolution of SCVs will facilitate better treatment and diagnosis of SCVs. We have previously reported that the slow growth of SCVs can be compensated for by mutations in the gene containing the original mutation responsible for the SCV phenotype (21). These intragenic mutations fully or almost fully restore the growth rate of SCVs but at the same time increase their susceptibility to aminoglycosides. Here, using selection by serial passage, we have shown the existence of two additional growth-compensatory pathways that are open to small colony variants. One path involves the acquisition of mutations that cause translational suppression of the SCV mutations (mutations of tRNAs, ribosomal protein S5, and RF-2), while the other path involves the acquisition of mutations in the genes *srrA* and *srrB* that encode the SrrAB two-component system. These mutations cause constitutive activation of a global transcriptional regulatory mechanism, SrrAB, and open up a route to increased ATP production that compensates for the slow-growth phenotype without the loss of resistance to aminoglycosides. A recent study has reported the existence of extragenic suppressors of the slow-growth phenotype of SCVs and hypothesized that the identified mutations were selected due to a constitutively activated general stress response (23).

The translational suppressor mutations compensate for the growth of SCVs by restoring the function of the mutated menadione and hemin biosynthesis genes that are responsible for the slow growth. They achieve this by nonsense suppression (via tRNAs, S5, and RF-2) or misincorporation of amino acids (via tRNAs). In each case, the improved growth was shown to be associated with restoration of the cross-membrane potential (Fig. 1). As with intragenic suppressor mutations, translational suppressor mutations increase susceptibility to aminoglycosides. For both the intragenic and translational suppressors, the kanamycin MICs of the compensated mutants, although reduced relative to the SCV, may remain higher (12 to 24 mg/liter) or become lower (0.19 to 0.75 mg/liter) than the MIC of the wild type (2 mg/liter). This variation in MIC may be explained by three factors: (i) not all translational suppressors (or intragenic mutations) restore the wild-type sequence, (ii) translational suppressors do not necessarily restore wild-type levels of protein, and (iii) mutations that affect the translation machinery may have global effects on translation. Thus, some of the nonsense suppressor mutations that were identified in this study are not predicted to restore the original amino acid sequence of the mutated proteins but would instead incorporate a different amino acid (Table S2). This was also observed for some of the intragenic suppressor mutations. In the case of intragenic suppressors, we observed that the kanamycin MIC did not always revert to the wild-type level (2 mg/liter) but varied between 0.75 and 8 mg/liter (Table S2). This variation in MIC is consistent with the idea that some of the mutant proteins do not have wild-type activity and thus do not restore the proton motive force to wild-type levels.

**FIG 5:**
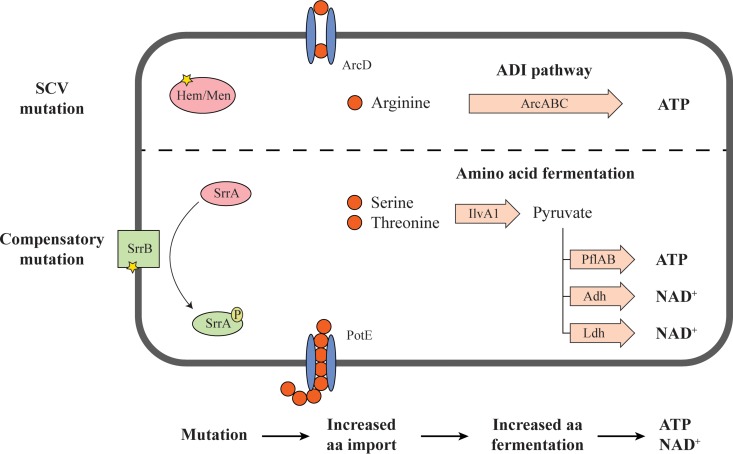
Cellular responses to SCV and transcriptional suppressor mutations. SCV-inducing mutations in the hemin or menadione biosynthesis pathways abolish cellular respiration. As a phenotypic compensation, genes involved in the arginine deiminase (ADI) pathway are upregulated, increasing the production of ATP from arginine to support growth. SCV suppressor mutations in *srrAB* cause constitutive activation of the SrrAB two-component system, which leads to increased expression of genes involved in peptide transport and in the fermentation of serine and threonine to produce ATP and NAD^+^.

Interestingly, the transcriptional suppressors (*srrAB*) increase the growth rate of SCVs without loss of resistance to aminoglycosides. SrrAB is a two-component transcriptional regulator that controls the expression of genes utilized during anaerobic respiration (40) and nitrosative stress resistance (28). Our data, in agreement with previous studies (36, 37) show that small colony variants of *S. aureus* that are incapable of producing ATP via respiration upregulate genes involved in the arginine deiminase (ADI) pathway that helps to ferment arginine to generate ATP (Fig. 5). The upregulation of the ADI pathway in SCVs may be an important explanation as to why SCVs are viable without a functional respiratory chain. The acquisition of SCV suppressor mutations in *srrA* or *srrB* leads to constitutive activation of the SrrAB regulatory system and upregulation of genes involved in the import and fermentation of the amino acids serine and threonine (Fig. 5). This enables the SCVs to utilize new carbon sources and thereby increase their growth. Since this mechanism does not restore the electron transport chain, it does not lead to an improved cross-membrane potential, and the cells therefore remain resistant to aminoglycosides.

In this study, we have described for the first time the functional basis of extragenic mutations that compensate for the slow growth of small colony variants of *S. aureus*. Intragenic and translational suppressor mutations lead to decreased resistance to aminoglycosides. Due to the decreased resistance, SCVs that acquire these types of compensatory mutations are less likely to survive treatment with aminoglycosides and are therefore more amenable to clinical therapy. The transcriptional suppressors, on the other hand, improve growth without affecting resistance to aminoglycosides. Additionally, activation of the SrrAB pathway has been shown to improve the protection of *S. aureus* from neutrophil killing, nitrosative stress, and hypoxia in the host (28, 41, 42). Due to these enhanced abilities, SCVs that evolve by the acquisition of transcriptional suppressor mutations have the potential to increase the clinical threat of small colony variants of *S. aureus*.

## MATERIALS AND METHODS

### Bacterial strains and growth conditions.

All mutant strains are derived from the antibiotic-susceptible *S. aureus* strain 8325-4 (43, 44). Bacteria were grown at 37°C in Luria-Bertani broth (LB) or on LA (LB medium plates solidified with 1.5% agar [Oxoid, Basingstoke, United Kingdom]). When required, kanamycin, hemin, menadione, or amino acids (Sigma-Aldrich, Stockholm, Sweden) were added to media at the concentrations stated in the text. The DNA cassette *srrAB*<>Em^r^ (45) was transduced into *S. aureus* 8325-4 by phage 80α-mediated transduction (46), and 80α lysates grown on that strain were then used to transduce the marker into menadione-auxotrophic SCVs.

### Isolation of small colony variants and growth-compensatory evolution.

Kanamycin was used to select SCVs. One hundred microliters of 80 independent overnight cultures of *S. aureus* 8325-4 were plated on LA plates containing 24 mg/liter of kanamycin. Plates were incubated at 37°C for up to 48 h. Small and extremely small colonies were picked and restreaked on the same selective media to purify. To select for growth-compensatory mutations, multiple independent cultures of genetically characterized SCV mutants were grown overnight in LB at 37°C and then serially passaged by transferring 2 µl of each overnight culture into 2 ml fresh medium with or without 15 mg/liter of kanamycin. After 3 to 15 cycles of passaging (30 to 150 generations), dilutions were plated on nonselective LA plates and incubated at 37°C for 48 h. Colonies of different sizes were picked, purified, and analyzed as described in the text.

### Auxotrophy complementation test.

Auxotrophy was determined by complementation with hemin and menadione. LA plates were spread with 5 × 10^5^ CFU of the strains to be tested. Six-millimeter-diameter filter discs (6 MM paper; GE Healthcare United Kingdom, Ltd., Little Chalfont, United Kingdom) were soaked in solutions of 1 g/liter (hemin and thymidine) or 200 mg/liter (menadione) and placed onto the inoculated LA plates. Plates were incubated at 37°C until an obvious lawn of growth was observed (approximately 24 h). A strong halo of thicker cell growth proximal to the cellulose disc was interpreted as a complementation of auxotrophy (4).

### Growth rate and final cell density measurements.

Exponential growth rates and final cell densities were measured in 300-µl cultures using a Bioscreen C machine (Oy Growth Curves Ab, Ltd., Finland). Colonies were picked from LA plates, with care taken to avoid large colonies, and were suspended in 500 µl LB (optical density at 600 nm [OD_600_] of ≈0.8). This dilution was then used to inoculate 300 µl fresh LB at a 1:100 dilution, which was then grown for 22 h at 37°C with continuous shaking in a honeycomb microtiter plate. Optical densities were measured at 600 nm at 4-min intervals, and the exponential growth rate was calculated from the increase in density as a function of time. The final cell density was defined as the optical density at 600 nm after 22 h of growth. When checking the ability of strains to use an amino acid as an extra carbon source, LB medium was supplemented with 25 mM of the appropriate amino acid. The pH of the medium supplemented with amino acids was adjusted to 7.3 ± 0.2.

### Genomic DNA preparation and whole-genome sequencing.

Bacteria were struck out on LA plates. When obvious colonies were observed, generally after 2 to 3 days of growth for SCVs, cells were scraped directly from the plates, with care to avoid heterogeneously sized colonies, and genomic DNA was prepared using the Qiagen genomic DNA buffer set and genomic-tip-100/G column (Qiagen, Hilden, Germany) according to the manufacturer’s specifications with one modification: lysostaphin (Sigma-Aldrich, Stockholm, Sweden) was added to buffer B1 at a final concentration of 100 mg/liter to aid lysis. Purified genomic DNA was sent to BGI (Tai Po, Hong Kong) for library assembly and genome sequencing. Genomic data were analyzed using the CLC Genomic Workbench 6 software (CLC Bio, Denmark).

### Preparation of RNA, RNA sequencing, and quantitative real-time PCR.

Bacterial colonies were scraped from plates and used to inoculate 5 ml fresh LB medium to an optical density at 600 nm (OD_600_) of approximately 0.05. Cultures were incubated with shaking at 37°C in 50-ml E-flasks. To ensure the cells were in exponential growth, samples of different genotypes were harvested at different time points after inoculation. Accordingly, wild-type strains were harvested after 150 min, menadione-auxotrophic SCVs were harvested after 300 min, and SCVs with *srrAB* amino acid substitutions or *srrAB* deletion mutations were harvested after 240 min and 340 min, respectively. Three milliliters of each culture was mixed with 6 ml RNAprotect (Qiagen, Hilden, Germany), and after 5 min of incubation at room temperature, cells were collected by centrifugation (4°C, 5,000 rpm, 12 min). Cell pellets were either sent to BGI (Tai Po, Hong Kong) for RNA preparation and sequencing, or alternatively, for qPCR, total RNA was isolated using the RNeasy minikit (Qiagen, Hilden, Germany) and transcribed to cDNA (High-capacity cDNA reverse-transcription kit; AB Applied Biosystems, USA) according to the manufacturer’s specifications. qPCR was performed as previously described (47) using *gyrB* and *gap* as reference genes (48). The oligonucleotides used for qPCR analysis are listed in Table S6 in the supplemental material.

10.1128/mBio.00358-17.8 TABLE S6  Oligonucleotides used for qPCR analysis. Download TABLE S6, PDF file, 0.1 MB. Copyright © 2017 Cao et al. 2017 Cao et al. This content is distributed under the terms of the Creative Commons Attribution 4.0 International license.

### MIC determinations.

MICs were determined on LA plates by using Etest strips (BioMérieux, Marcy l’Étoile, France) according to the manufacturer’s instructions.

### Cross-membrane potential measurement.

Bacteria were grown with shaking at 37°C until the exponential growth phase. Cultures were diluted to approximately 1 × 10^6^ CFU/ml in filtered phosphate-buffered saline (PBS), and DiOC_2_ (Sigma-Aldrich, Stockholm, Sweden) was added to a final concentration of 30 µM. Depolarized control samples were supplemented with CCCP (Sigma-Aldrich, Stockholm, Sweden) to a final concentration of 1 µM. After 15 min of incubation at 37°C, the florescence intensities of green fluorescent protein (GFP) and Texas Red were measured with a MACSQuant VYB (Miltenyi Biotec, Inc., Bergisch Gladbach, Germany). The ratio of red over green fluorescence was used to determine the membrane potential of each strain (49).

### Cellular ATP measurements.

Cellular ATP levels were measured using BacTiter-Glo microbial cell viability assay (Promega). Bacteria were grown with shaking at 37°C until the exponential growth phase. One hundred microliters of each culture was mixed with 100 µl of BacTiter-Glo reagent, the mixture was incubated at room temperature for 5 min, and the luminescence of each sample was measured. A medium-only sample was used to obtain a value for background luminescence. A viable cell count was performed for each sample to calculate the ATP production per cell.
