# Supplementary figures and images for: Alternative Evolutionary Pathways for Drug-Resistant Small Colony Variant Mutants in Staphylococcus aureus
Source: mBio. 2017 Jun 20;8(3):e00358-17. doi: 10.1128/mBio.00358-17 (PMC5478891; doi:10.1128/mBio.00358-17)

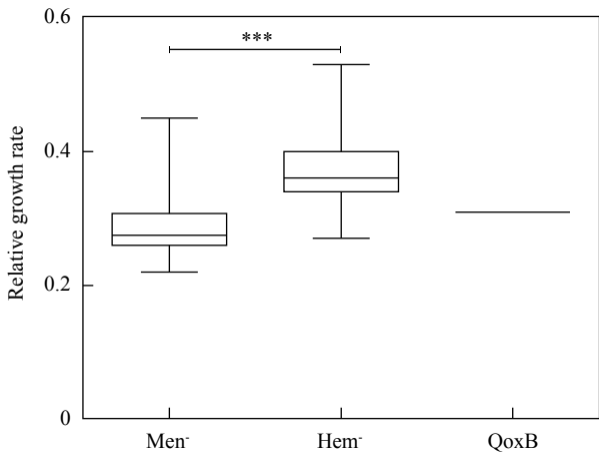

Supplement: FIG S1 [file mbo003173349sf1.pdf]

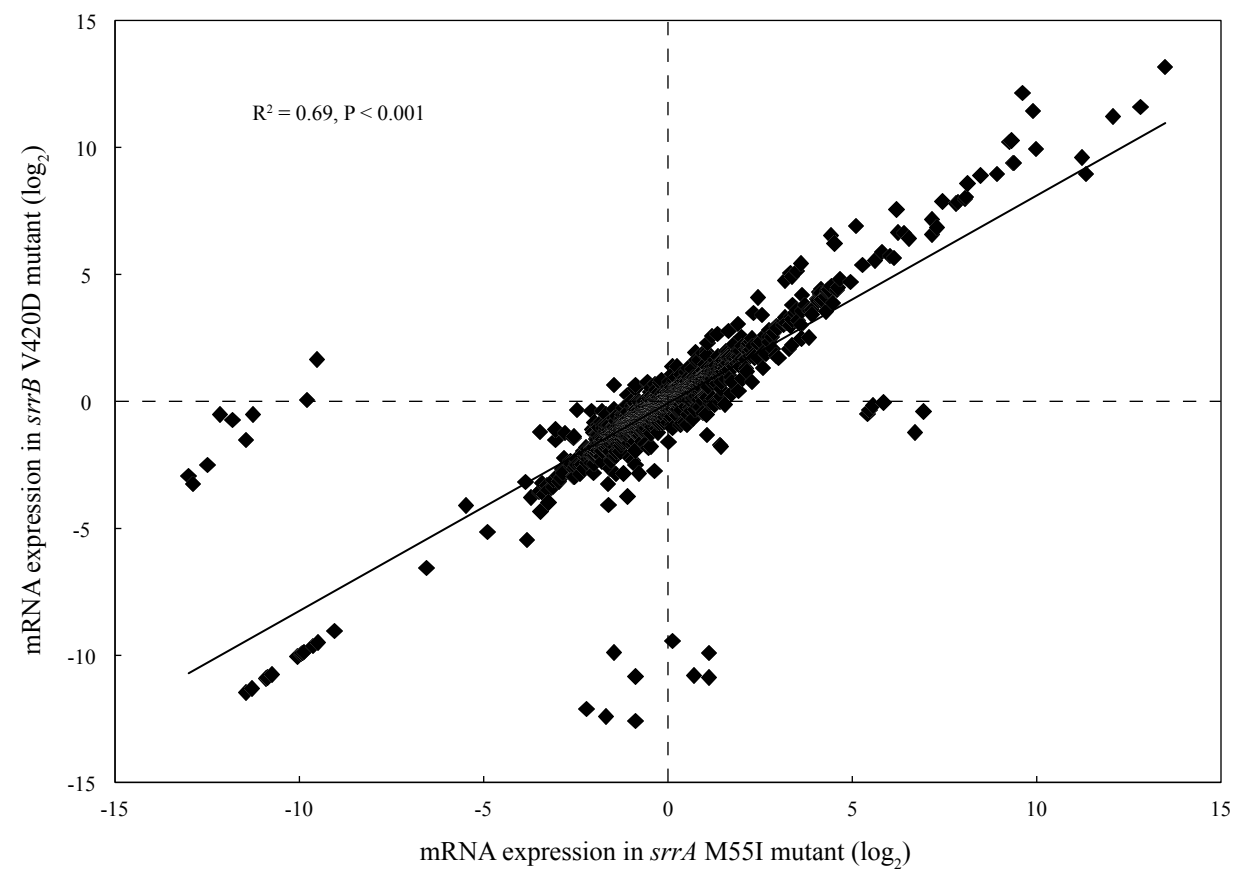

Supplement: FIG S2 [file mbo003173349sf2.pdf]
