# Supplementary material for: Alternative Evolutionary Pathways for Drug-Resistant Small Colony Variant Mutants in Staphylococcus aureus
Source: mBio. 2017 Jun 20;8(3):e00358-17. doi: 10.1128/mBio.00358-17 (PMC5478891; doi:10.1128/mBio.00358-17)
Supplement: TABLE S1 [file mbo003173349st1.pdf]

Table S1. Genotypes and phenotypes of 26 whole genome sequenced SCVs.

[a]Strain	[b]SCV mutations	[b]Additional mutations	Auxotrophy	[c]Fitness ± SD	Kan MIC
AH610	Wild-type	-	-	1.00	2
AH866	*hemB* P164L	*fusA* S586P	Hem$^-$	0.34 ± 0.02	192
AH870	*hemB* G275R	*fusA* G544D	Hem$^-$	0.30 ± 0.01	128
AH867	*hemC* GA→TT<br>(RBS nts -12/11)	*fusA* T150I<br>*topB* Q211*	Hem$^-$	0.34 ± 0.02	64
AH877	*hemC* Q17K	*fusA* H572Y	Hem$^-$	0.36 ± 0.00	192
AH671	*hemC* E70fs	-	Hem$^-$	0.40 ± 0.02	32
AH646	*hemC* D74Y	-	Hem$^-$	0.44 ± 0.00	32
AH727	*hemC* D104Y	-	Hem$^-$	0.42 ± 0.00	32
AH862	*hemD* Q107fs	-	Hem$^-$	0.30 ± 0.01	32
AH839	*hemE* A211fs	SAOUHSC-00468 A63E	Hem$^-$	0.36 ± 0.01	48
AH868	*hemH* K158*	-	Hem$^-$	0.35 ± 0.00	48
AH690	*menA* Q7*	-	Men$^-$	0.32 ± 0.01	48
AH847	*menA* W15*	SAOUHSC-00698 L319S	Men$^-$	0.29 ± 0.01	48
AH654	*menA* D205E	SAOUHSC-01014 R366L	Men$^-$	0.27 ± 0.00	32
AH704	*menA* E72K	*purH* L302F	Men$^-$	0.28 ± 0.01	64
AH664	*menA* D81N	SAOUHSC-00885 H42fs	Men$^-$	0.23 ± 0.04	64
AH1341	*menA* G87R	-	Men$^-$	0.32 ± 0.01	24
AH864	*menA* Q180*	*glpK* G349C	Men$^-$	0.27 ± 0.01	48
AH680	*menA* D205H	SAOUHSC-00854 R145Q	Men$^-$	0.26 ± 0.01	24
AH631	*menB* G81*	*qoxA* M57K	Men$^-$	0.25 ± 0.01	64
AH846	*menB* D98G	SAOUHSC-02417 A114S	Men$^-$	0.27 ± 0.01	48
AH855	*menB* D98G	*fusA* W120L<br>SAOUHSC-02417 A114S	Men$^-$	0.27 ± 0.02	96
AH841	*menB* G121D	-	Men$^-$	0.29 ± 0.01	64
AH875	*menB* D151N	-	Men$^-$	0.29 ± 0.01	48
AH878	*menB* D229A	*rplF* M98fs	Men$^-$	0.26 ± 0.01	256
AH635	*menE* Y31*	-	Men$^-$	0.28 ± 0.01	32
AH1342	-	*qoxB* G57D<br>*srrA* V113F and K114fs<br>*oxaA* K164I (SAOUHSC-02327)<br>*ftsQ* V216F (SAOUHSC-01148)	-	0.26 ± 0.01	64

[a] All mutant strains are isogenic to the wild-type AH610 (*S. aureus* 8325-4).
[b] RBS indicates the mutation occurs in the putative ribosome binding site of *hemC*, asterisk and fs indicate that the mutation creates a nonsense codon or a frameshift mutation in the coding sequence of the gene.
[c] Relative fitness is measured as exponential growth rate in LB ± standard deviation (wild type is set to 1)
