# Supplementary material for: Alternative Evolutionary Pathways for Drug-Resistant Small Colony Variant Mutants in Staphylococcus aureus
Source: mBio. 2017 Jun 20;8(3):e00358-17. doi: 10.1128/mBio.00358-17 (PMC5478891; doi:10.1128/mBio.00358-17)
Supplement: TABLE S2 [file mbo003173349st2.pdf]

Table S2. Genotypes and phenotypes of SCVs and growth-compensated mutants after selection.

		Acquired mutations present after experimental evolution					[a]Growth phenotype		MIC
**Strain**	**SCV genotype**	**Intragenic mutations**	**[b]Translational mutations**	**Transcriptional mutations**	**Other mutations**	**[c]N**	**Rate**	**Yield**	**KAN**
**AH610**	**Wild-type**						**1.00**	**1.00**	**2**
**AH671**	***hemC* E70fs**						**0.40 ± 0.02**	**0.27 ± 0.02**	**32**
AH879		Reversion to wild-type	-	-	-	10/10	0.97 ± 0.01	0.98 ± 0.00	1
**AH646**	***hemC* D74Y**						**0.42 ± 0.02**	**0.31 ± 0.00**	**32**
AH1104		*hemC* D74C	-	-	-	10/12	0.95 ± 0.01	0.98 ± 0.02	3
AH1169		*hemC* D74H	-	-	-	1/12	0.76 ± 0.01	0.95 ± 0.01	8
AH1181		*hemC* D74S	-	-	-	1/12	0.87 ± 0.01	1.00 ± 0.01	2
**AH727**	***hemC* D104Y**						**0.42 ± 0.00**	**0.27 ± 0.00**	**64**
AH1294		Reversion to wild-type	-	-	-	3/22	1.01 ± 0.02	0.99 ± 0.01	1.5
AH1114		*hemC* D104C	-	-	-	19/22	0.98 ± 0.02	1.01 ± 0.01	0.75
**AH868**	***hemH* K158^{UAA}**						**0.43 ± 0.02**	**0.26 ± 0.01**	**32**
AH1098		Reversion to wild-type	-	-	-	1/20	0.99 ± 0.03	0.99 ± 0.01	2
AH1212		*hemH* K158E	-	-	-	1/20	0.83 ± 0.05	0.99 ± 0.01	1.5
AH1243		*hemH* K158L	-	-	-	3/20	0.88 ± 0.04	0.98 ± 0.01	1.5
AH1083		*hemH* K158Y	-	-	-	4/20	0.89 ± 0.01	1.02 ± 0.01	1.5
AH1074			tRNA-ser T00048	-	SAOUHSC-00811 A161T	1/20	0.65 ± 0.01	0.80 ± 0.02	1

			(AGU to AUU)						
AH1078			tRNA-ser T00048 (AGU to AUU)	-	-	1/20	0.59 ± 0.02	0.78 ± 0.00	1.5
AH1255			tRNA-ser T00048 (AGU to AUU)	-	*dnaK* D221E	1/20	0.69 ± 0.00	0.83 ± 0.01	0.75
AH1358			tRNA-ser T00051 (AGU to AUU)	-	SAOUHSC-01715 T175S	1/20	0.49 ± 0.01	0.62 ± 0.04	0.5
AH1080			tRNA-Tyr T00057 (AUG to AUU)	-	-	1/20	0.66 ± 0.01	0.93 ± 0.01	4
AH1071			tRNA-Tyr T00057 (AUG to AUU)	-	SAOUHSC-00908 F265I<br>*trpA* P172T<br>*aroA* E198D	1/20	0.66 ± 0.01	0.93 ± 0.01	4
AH1091			tRNA-Tyr T00058 (AUG to AUU)	-	SAOUHSC-01267 V11F	1/20	0.72 ± 0.01	0.92 ± 0.01	3
AH1095			tRNA-Tyr T00058 (AUG to AUU)	-	-	1/20	0.71 ± 0.01	0.90 ± 0.01	3
AH1088			tRNA-Tyr T00058 (AUG to AUU)	-	-	1/20	0.73 ± 0.01	0.92 ± 0.00	2
AH1064			tRNA-Tyr T00058 (AUG to AUU)	-	SAOUHSC-00484 Q391E	1/20	0.71 ± 0.01	0.91 ± 0.01	2
AH1357			*rpsE* V105F	-	-	1/20	0.26 ± 0.01	0.35 ± 0.01	8
**AH847**	***menA* W15^{UGA}**<br>**SAOUHSC-00698 L319S**						**0.39 ± 0.07**	**0.29 ± 0.00**	**48**
AH1364			*prfB* G118S	-	-	1/10	0.41 ± 0.07	0.50 ± 0.09	1.5

AH1368			*prfB* R133H	-	-	1/10	0.39 ± 0.01	0.42 ± 0.02	24
AH1365			*prfB* stop codon[f]	-	-	1/10	0.63 ± 0.02	0.83 ± 0.01	2
**AH855**	***menB* D98G *fusA* W120L SAOUHSC-02417 A114S**						**0.22 ± 0.00**	**0.16 ± 0.01**	**96**
AH1379		*menB* G81V	-	-	-	1/10	0.23 ± 0.00	0.59 ± 0.04	8
AH1380		*menB* D98S	-	-	-	2/10	0.48 ± 0.00	0.82 ± 0.01	3
AH1384		*menB* D240A	-	-	-	1/10	0.49 ± 0.01	0.79 ± 0.02	6
**AH875**	***menB* D^{GAU}151N^{AAU}**						**0.29 ± 0.01**	**0.22 ± 0.01**	**48**
AH1166		Reversion to wild-type				20/34	0.98 ± 0.04	0.99 ± 0.01	1.5
AH1024		*menB* Q100E				6/34	0.95 ± 0.01	0.97 ± 0.00	1.5
AH1037			tRNA-Asp T00011 (CUG to UUA)	-	-	1/34	0.29 ± 0.01	0.43 ± 0.02	24
AH1011			tRNA-Asp T00012 (CUG to UUA)	-	SAOUHSC-01252 E316*	1/34	0.23 ± 0.01	0.58 ± 0.03	2
AH1208			-	*srrA* M55I	SAOUHSC-02481 T131K	1/34	0.48 ± 0.02	0.25 ± 0.01	32
AH1203			-	*srrB* R316P	SAOUHSC-00435 F223S	1/34	0.46 ± 0.01	0.26 ± 0.00	32
AH1201			-	*srrB* S368L	-	1/34	0.47 ± 0.01	0.25 ± 0.01	48
AH1197			-	*srrB* S368L	SAOUHSC-00136 L75I	1/34	0.48 ± 0.01	0.25 ± 0.00	32
AH1131			-	*srrB* V420D	SAOUHSC-01906 G144S	1/34	0.48 ± 0.02	0.25 ± 0.01	48
AH1199			-	*srrB* V420F	*narH* S1017L SAOUHSC-02907 K20*	1/34	0.44 ± 0.03	0.26 ± 0.01	24
**AH635**	***menE* Y31^{UAA}**						**0.29 ± 0.02**	**0.21 ± 0.00**	**32**

AH962		Reversion to wild-type		-	-	2/22	1.00 ± 0.02	1.00 ± 0.01	2
AH1139		*menE* Y31E		-	-	2/22	0.96 ± 0.04	0.97 ± 0.01	1.5
AH923		*menE* Y31L		-	-	2/22	0.96 ± 0.02	0.99 ± 0.01	1.5
AH1345			tRNA-Gln T00016 (GUU to AUU)	-	-	1/22	0.59 ± 0.05	0.83 ± 0.00	0.75
AH1347			tRNA-Gln T00016 (GUU to AUU)	-	-	1/22	0.61 ± 0.04	0.85 ± 0.04	1.5
AH1145			tRNA-Gln T00016 (GUU to AUU)	-	tRNA-Gln T00016 nt C41U	1/22	0.83 ± 0.04	0.91 ± 0.01	1.5
AH939			tRNA-Gln T00017 (GUU to AUU)	-	tRNA-Lys T00037 nt G53A	1/22	0.75 ± 0.02	0.85 ± 0.02	0.38
AH1133			tRNA-Glu T00019 (CUU to AUU)	-	-	1/22	0.47 ± 0.00	0.51 ± 0.05	2
AH1351			tRNA-Leu T00030 (AAU to AUU)	-	-	1/22	0.43 ± 0.01	0.61 ± 0.10	0.19
AH994			tRNA-Lys T00037 (UUU to AUU)	-	-	1/22	0.58 ± 0.01	0.82 ± 0.02	2
AH915			tRNA-Lys T00035 (UUU to AUU)	-	-	1/22	0.53 ± 0.01	0.72 ± 0.04	1.5
AH936			tRNA-Lys T00036 (UUU to AUU)	-	*fbp3* E6K	1/22	0.57 ± 0.01	0.83 ± 0.03	2
AH919			tRNA-Ser T00048 (AGU to AUU)	-	-	1/22	0.63 ± 0.05	0.86 ± 0.02	1

AH927			tRNA-Ser T00052 (AGU to AUU)	-	-	1/22	0.44 ± 0.01	0.62 ± 0.08	0.25
AH930			tRNA-Ser T00052 (AGU to AUU)	-	-	1/22	0.46 ± 0.08	0.63 ± 0.16	0.25
AH1143			tRNA-Ser T00052 (AGU to AUU)	-	SAOUHSC-01358 T198I SAOUHSC-00994 V48F	1/22	0.51 ± 0.01	0.63 ± 0.04	0.38
AH1149			tRNA-Tyr T00057 (AUG to AUU)	-	-	1/22	0.85 ± 0.02	0.90 ± 0.00	2
AH1152			tRNA-Tyr T00057 (AUG to AUU)	-	*agrA* Y123N SAOUHSC-02153 T24F *corC* R412S	1/22	0.80 ± 0.08	0.95 ± 0.01	2
AH910			tRNA-Tyr T00058 (AUG to AUU)	-	-	1/22	0.88 ± 0.03	0.92 ± 0.01	2

[a] Growth phenotype.
Exponential growth rate relative to that of the wild type ± standard deviation.
Final optical density of overnight cultures relative to that of the wild type ± standard deviation.

[b] Acquired mutations affecting the translation machinery.
All tRNA nonsense (UAA) suppressor mutations change the tRNA anticodon to 3'-UUA-5'.
All tRNA missense ($D151N^{AAU}$) suppressor mutations change the tRNA anticodon to 3'-AUU-5'.
T000XX notation represents individual tRNA gene numbers as annotated in the *S. aureus* genome.
The single nt deletion in the *prfB* stop codon of AH1365 leads to a predicted 22 amino acid extension of the protein.

[c] N is number of mutants independently evolved from each SCV that had acquired the genotype listed. For two of the strains (AH847, AH855) the evolved very fast-growing strains were not analysed further.
