# Supplementary material for: Alternative Evolutionary Pathways for Drug-Resistant Small Colony Variant Mutants in Staphylococcus aureus
Source: mBio. 2017 Jun 20;8(3):e00358-17. doi: 10.1128/mBio.00358-17 (PMC5478891; doi:10.1128/mBio.00358-17)
Supplement: TABLE S3 [file mbo003173349st3.pdf]

Table S3. Relative gene expression measured by qPCR, comparing the affect of SCV-suppressor mutations in *srrAB* with the affect of a deletion of *srrAB*.

Gene number, name	Gene function	mRNA level relative to SCV AH875 (log2 ratio) ± S.D		
		srrA **M55I (AH1208)**	***srrB*** **V420D (AH1131)**	**Δ*srrAB*** **(AH1670)**
SAOUHSC_00113, *adhE*	Alcohol dehydrogenase	2.84 ± 0.53	2.73 ± 0.3	-1.64 ± 0.24
SAOUHSC_00187, *pflB*	Formate acetyltransferase	3.13 ± 0.92	2.9 ± 0.48	-3.72 ± 0.53
