# Supplementary material for: Alternative Evolutionary Pathways for Drug-Resistant Small Colony Variant Mutants in Staphylococcus aureus
Source: mBio. 2017 Jun 20;8(3):e00358-17. doi: 10.1128/mBio.00358-17 (PMC5478891; doi:10.1128/mBio.00358-17)
Supplement: TABLE S5 [file mbo003173349st5.pdf]

Table S5. MICs of populations with SCV-suppressor mutations in *srrAB* after 30 generations of evolution.

Parental strain	Evolved lineage	SCV mutation	*srrAB*	Kan MIC
AH1560		*menB* D98G	*srrB* T373K	48
	1	*menB* D98G	*srrB* T373K	64
	2	*menB* D98G	*srrB* T373K	48
	3	*menB* D98G	*srrB* T373K	48
	4	*menB* D98G	*srrB* T373K	48
	5	*menB* D98G	*srrB* T373K	48
AH1208		*menB* D151N	*srrA* M55I	32
	1	*menB* D151N	*srrA* M55I	48
	2	*menB* D151N	*srrA* M55I	48
	3	*menB* D151N	*srrA* M55I	32
	4	*menB* D151N	*srrA* M55I	32
	5	*menB* D151N	*srrA* M55I	32
AH1201		*menB* D151N	*srrB* S368L	48
	1	*menB* D151N	*srrB* S368L	48
	2	*menB* D151N	*srrB* S368L	32
	3	*menB* D151N	*srrB* S368L	32
	4	*menB* D151N	*srrB* S368L	48
	5	*menB* D151N	*srrB* S368L	32
AH1131		*menB* D151N	*srrB* V420D	48
	1	*menB* D151N	*srrB* V420D	48
	2	*menB* D151N	*srrB* V420D	48
	3	*menB* D151N	*srrB* V420D	48
	4	*menB* D151N	*srrB* V420D	48
	5	*menB* D151N	*srrB* V420D	48
AH1643		*hemE* A211fs	*srrB* A250T	48
	1	*hemE* A211fs	*srrB* A250T	64
	2	*hemE* A211fs	*srrB* A250T	48
	3	*hemE* A211fs	*srrB* A250T	64
	4	*hemE* A211fs	*srrB* A250T	48
	5	*hemE* A211fs	*srrB* A250T	48
