# Supplementary material for: Alternative Evolutionary Pathways for Drug-Resistant Small Colony Variant Mutants in Staphylococcus aureus
Source: mBio. 2017 Jun 20;8(3):e00358-17. doi: 10.1128/mBio.00358-17 (PMC5478891; doi:10.1128/mBio.00358-17)
Supplement: TABLE S6 [file mbo003173349st6.pdf]

Table S6. Oligonucleotides used for qPCR analysis.

Gene	Forward oligonucleotide (5’ – 3’)	Reverse oligonucleotide (5’ – 3’)
adhE	AAGCGTGCAGCAGAAGTTG	GACTTAACCATGCCCGAACC
gap	AGTGAACCAGATGCAAGCAAATTAC	TTTTAGCGCCTGCTTCAATATG
gyrB	CCAGGTAAATTAGCCGATTGC	AAATCGCCTGCGTTCTAGAG
pflB	CGAAGGCGACTTCCCTAAA	GCTTTACGTCCGTCTGGTGT
